# Mn/Ce Oxides Decorated Polyphenylene Sulfide Needle-Punching Fibrous Felts for Dust Removal and Denitration Application

**DOI:** 10.3390/polym12010168

**Published:** 2020-01-09

**Authors:** Ying Chen, Hongwei He, Shaohua Wu, Xin Ning, Fuxing Chen, Yanru Lv, Juan Yu, Rong Zhou

**Affiliations:** 1 Industrial Research Institute of Nonwovens & Technical Textiles, College of Textiles & Clothing, Qingdao University, Qingdao 266071, China; cheny06301@163.com (Y.C.); hhwpost@163.com (H.H.); xning@qdu.edu.cn (X.N.); fuxing1991@gmail.com (F.C.);; 2 College of Textiles & Clothing, Qingdao University, Qingdao 266071, China; shaohua.wu@qdu.edu.cn

**Keywords:** catalyst loading, sodium alginate deposition, plasma treatment, denitration, filtration efficiency

## Abstract

Development of a novel filter material is urgently required for replacing the high-cost flue gas purification technology in the simultaneous removal of both fine dust and Nitrogen oxides (NO_x_). In this study; polyphenylene sulfide (PPS) needle-punching fibrous felts (NPFF) were employed as the filter material to remove the fine dust; and in the meanwhile; Mn and Ce oxides were loaded onto the PPS NPFF as the catalyst for selective catalytic reduction of NO_x_ with NH_3_. Two different pretreatment methods; i.e., sodium alginate (SA) deposition and plasma treatment; were employed to modify the PPS NPFF before the traditional impregnation and thermal treatment processes during the catalyst loading. The results showed that these two pretreatment methods both afforded the PPS NPFF with the enhanced loading rate and stability of Mn/Ce oxides compared to those without any pretreatments; which were significantly beneficial for the denitration application. Moreover; we found that both SA deposition and plasma pre-treated samples presented excellent dust-removal properties; and the filtration efficiency could reach 100% when the particle size of the fine particulates was above 4 μm. This study demonstrated that our Mn/Ce oxides decorated PPS NPFF have great potential to be applied in the fuel gas purification field; due to their stable structure; handling convenience; and excellent filtration efficiency; as well as high denitration performance.

## 1. Introduction

Environmental pollution is a combined effect of industrialization and urbanization, resulting in a significant decline in the human health index [1,2]. Recently, the rapid increase of energy consumption directly spurred the enhanced emissions of various pollutants during the combustion of solid fuels. It is predicted that the total consumption of solid fuels will increase by roughly 20% from 2015 to 2035 [3]. Fine particles are one of the key pollutant components from exhaust emissions. In order to address the pollution issues induced by fine particulates, bag filter technology has been widely employed by industry [4], which is applied into the field of high temperature and high concentration flue gas treatment [5,6]. 

Except for dust removal, another important aspect for flue gas processing is to effectively get rid of the hazard nitrogen oxide (NO_x_) component, which could lead to a wide variety of respiratory diseases, such as pneumonia and bronchitis [7]. Presently, ammonia selective catalytic reduction (NH_3_-SCR) technology is recognized as the most effective way to realize the removal of NO_x_ existing in the fuel-burning exhaust gas [8]. NH_3_-SCR technology takes NH_3_ as the reducing agent to reduce toxic NO_x_ to non-toxic N_2_ and H_2_O under the action of a catalyst [9]. With the low cost, high efficiency and easy promotion, NH_3_-SCR technology has become a hot subject of extensive studies in the past few years [10,11].

Catalyst performance has been demonstrated to be a dominant factor that notably affects the NO_x_ reduction efficiency of SCR technology [12]. Manganese (Mn)-based catalysts have attracted much attention because of their low production cost and excellent catalytic activity at relatively low temperature (100–250 °C). Peña et al. employed TiO_2_ as supportive material and compared the catalytic performance of several different transition metal oxides [13]. The catalytic activity was found in the following order under the operating temperature of 120 °C: Mn > Cu > Cr > Co > Fe > V > Ni. Other studies indicated that the catalyst sintering phenomenon was effectively reduced, and the thermal stability and service life of the catalyst were greatly improved, when other metal elements were properly doped into Mn-based catalysts [14,15]. Ceric oxide (CeO_2_) was verified to effectively increase the stability of the metal oxide catalyst during the loading process and obviously enhance denitration efficiency during the NH_3_-SCR process [16,17]. 

In order to optimize the structure of the filtration system, and reduce the equipment and operating cost of enterprise, the device that can achieve the joint removal of multiple pollutants is becoming the main development orientation in the future flue gas purification field [18,19]. In this case, this study aims to develop a new filter material that combines the features of the traditional bag filter and NH_3_-SCR technology to provide both dust removal and denitrification functions. Polyphenylene sulfide (PPS) needle-punching fibrous felts (NPFF) were employed as both dust-removal substrate and catalyst-supporting materials, because previous studies demonstrated that PPS NPFF possessed excellent heat and chemical resistances [20,21]. Mn and Ce-based catalysts were loaded on the PPS NPFF through three steps, i.e., pretreatment, impregnation and thermal treatment. Two different pretreatment methods, i.e., sodium alginate (SA) deposition and plasma treatment, were used to investigate how the pretreatment method affects the loading rate and stability of catalysts. This work also designed and implemented a laboratory-specific NH_3_-SCR denitration test device to simulate the high-temperature working environment in practical applications. The effect of pretreatment method on the denitration performance of the as-prepared Mn/Ce oxides decorated PPS NPFF was studied, together with the changes of morphology, microstructure, and mechanical property of the PPS NPFF before and after the catalyst loading process. We also characterized and compared the dust removal properties of the finally-obtained Mn/Ce oxides decorated PPS NPFF manufactured by different preparation methods.

## 2. Experimental

### 2.1. Materials

PPS NPFF (Gram weight: 580 g/m^2^, Thickness: 2.9 mm) were supplied by Xinli Environmental Protection Materials Co., Ltd. (Zibo, Shandong, China). Sodium alginate (SA, AR), manganese nitrate (Mn(NO_3_)_2_, AR) and cerium nitrate (Ce(NO_3_)_3_, AR) were purchased from Dingshengxin Chemical Co. Ltd. (Tianjin, China), Shanpu Chemical Co. Ltd. (Shanghai China), and Qingdao Haizhilin Co. Ltd. (Shandong, China), respectively. Nitric oxide (NO) and ammonia (NH_3_) were both provided by Qingdao Three-factors Gas Technology Co. Ltd. (Qingdao, Shandong, China). Compressed air without water (H_2_O) was obtained from Qingdao Haide Gas Co. Ltd. (Qingdao, Shandong, China).

### 2.2. Catalyst Loading Methods

All PPS NPFF were cut into 20 × 20 cm squares, and utilized as the catalyst supporting materials in the present work. A traditional impregnation method in combination with a subsequent thermal treatment process was employed to synthesize and load Mn and Ce-based catalysts onto the PPS NPFF. Specifically, PPS NPFF were immersed into several different Mn (NO_3_)_2_ and Ce (NO_3_)_3_ mixed solutions at room temperature. After the predetermined time point, the Mn (NO_3_)_2_ and Ce (NO_3_)_3_ coated PPS NPFF were taken out and placed in a drying oven, experiencing thermal treatment at a predetermined temperature for preset times. In order to obtain the optimal loading efficiency and stability of catalysts, we optimized the parameters for both impregnation and thermal treatment processes. The detailed information is shown in the Supplementary Information. The optimal Ce molality and Mn/Ce molar ratio of impregnation solution, impregnation time, temperature and time of thermal treatment were found to be 0.07 mol/L, 6/1, 60 min, 200 °C, and 60 min, respectively. The samples made by using these optimal parameters were named as PPS NPFF/Mn/Ce-C, which were defined to be control groups.

**Figure 1:**
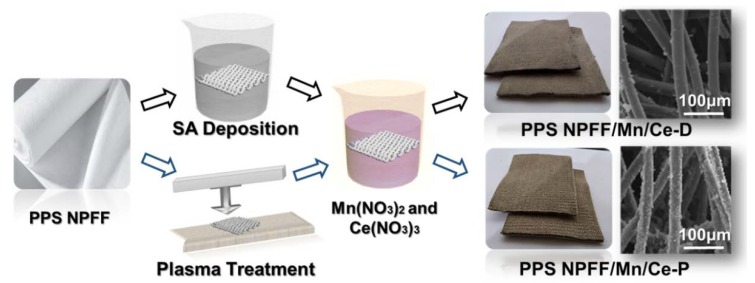
Schematic of the fabrication of Mn/Ce oxides decorated polyphenylene sulfide (PPS) needle-punching fibrous felts (NPFF).

To further improve the catalyst loading rate, two different pretreatment methods, i.e., SA deposition and plasma treatment, were utilized to modify the PPS NPFF before the impregnation and thermal treatment processes (Figure 1). Alginate is a natural polysaccharide, produced from seaweed and paid attention to due to its multifarious properties, efficiency, and affordable price, non-biotic and non-biodegradable nature [22]. As an excellent cationic heavy metal adsorbent, the molecular backbone of sodium alginate contains a large number of highly active carboxylic ions (-COO-) and hydroxyl (-OH) groups [23]. Creating a matrix of alginate on the surface of compounds caused exceptional properties, a high adsorbent density, and a high surface area as well as increased adsorption capability [24,25]. Thus, SA deposition employed SA as a binder for catalyst immobilization. PPS NPFF was immersed into several different SA solutions at room temperature. After the predetermined time point, the SA coated PPS NPFF were taken out and dried in a drying oven at 60 °C for 24 h. The as-obtained SA coated PPS NPFF further experienced the impregnation and thermal treatment processes by using the same parameters with PPS NPFF/Mn/Ce-C fabrication. We designed two single factor experiments to optimize the experimental parameters of SA deposition. Detailed information is shown in the Supplementary Information. The optimal mass fraction of SA was 0.8%, and the optimal immersion time was 60 min. The samples made by using the optimal parameters of SA deposition were named as PPS NPFF/Mn/Ce-D.

We also employed low-temperature plasma technology to pretreat the PPS NPFF, which could etch the surface of the felt and increase the bonding performance of the felt surface [26,27]. The applicability of PPS fibers in catalyst decorating is affected because of their compact structure, smooth surface, and poor friction properties. The surface of PPS fiber has been etched, crosslinked, group introduced, roughened, etc., due to highly excited and unstable active particles in air plasma. On the surface of PPS fibers, fine cracks are formed, convex deposits are generated, and a series of polar groups containing oxygen and sulfur are generated [28,29,30,31]. Therefore, the air plasma increases the micro-roughness of the fiber surface, improves the fiber adhesion, biocompatibility, etc., and further facilitates the decorating of the catalyst. Plasma treatment was carried out by using the Atmospheric Pressure Plasma System Model AS400+PFW10 (Plasma Treat GmbH, Steinhagen, Germany). The air was used to generate plasma, and the gas flow rate of the discharge and the jet-sample-distance were 1000 L/h and 3 cm, respectively. In addition, there were four other main parameters, i.e., voltage, duty ratio, jet moving speed, frequency, which could significantly affect the treatment results. Therefore, a series of single factor experiments and even orthogonal experiments were utilized to obtain the optimal parameters, as shown in the Supplementary Information. The optimal plasma voltage, jet moving speed, duty ratio and frequency were insured to be 280 V, 8 m/min, 60% and 19 KHz. The plasma-treated PPS NPFF further experienced the impregnation and thermal treatment processes by utilizing the same parameters with PPS NPFF/Mn/Ce-C fabrication. The samples made by using optimal parameters of plasma treatment were named as PPS NPFF/Mn/Ce-P. 

### 2.3. Catalyst Loading Rate and Fastness Characterization

Catalyst loading rate was defined as the ratio of the increased weight after catalyst loading to the weight of original sample. It was calculated as follows:

(1) $$R=\frac{M_{2}-M_{1}}{M_{1}}\times 100$$

where: R: loading rate, %; M_1_: the weight of original sample, g; M_2_: the weight of the sample after catalyst loading, g.

An air blower (Philips, Amsterdam, NY, USA) was employed to test the catalyst loading fastness. The samples after the catalyst loading were placed under a 2000 mL/min air flow for 5 h. The catalyst loading fastness was defined as the ratio of the weight of the sample after the airflow process to the weight of the original sample. It was calculated as follows:

(2) $$Q=\frac{M}{M_{0}}\times 100$$

where: Q: loading fastness, %; M: sample weight after the airflow process, g; M_0_: the weight of the original sample, g.

### 2.4. Denitration Measurement

**Figure 2:**
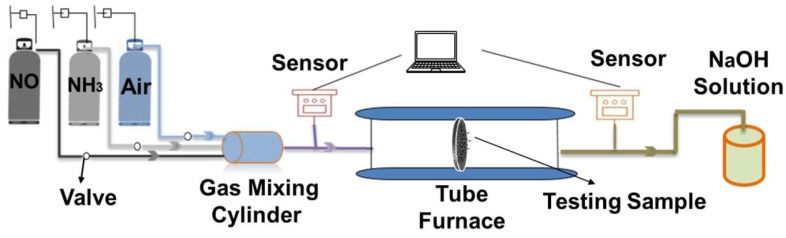
Schematic illustration of our home-made ammonia selective catalytic reduction (NH_3_-SCR) denitration testing device.

A home-made NH_3_-SCR denitration testing device (Figure 2) was designed and implemented by our group, to better mimic the practical denitration processing in the thermal power plant. The three different Mn/Ce Oxides loaded PPS NPFF samples were cut into a circular shape with a diameter of 10 cm, and further placed into a tube furnace with the total volume of 4.6 L. The whole device was connected with the silicone hoses to avoid the gas leakage. Before test, the compressed air was firstly introduced to keep the gas environment inside the channel in a normal air state. Then, the compressed air, NH_3_ and NO were continuously introduced into the channel, and the concentrations of each gas component in the inlet and outlet were monitored by the sensors in site. The total gas flow was maintained as 140 mL/min, and the compressed air flow was maintained at 120 mL/min. The range of NH_3_/NO flow ratio was 0.8–1.6. After the sensor display was stabilized, the tube furnace started to heat up. The pre-set testing temperature ranged from 80 °C to 220 °C. The sensor displays were recorded in a real time manner by using an intelligent test system (Shenwei Electronic Technology Co., Ltd., Shanghai, China) connected to a computer.

The denitration performance of test samples was indicated by the denitration rate. The calculation formula is as follows:

(3) $$\eta \;=\;\frac{W_{1}-W_{2}}{W_{1}}\times 100$$

where: η: denitrification rate, %; W_1_: the NO concentration at inlet, ppm; W_2_: the NO concentration at outlet, ppm.

### 2.5. Air Permeability and Filtration Characterization

The air permeability of the samples was measured according to the ISO 9237 (1995) standard test method by using a Frazier Air Permeability Tester (YG461E, NBFY Co. Ltd., Ningbo, China) with a fixed testing area of 20 cm^2^ and the pressure drop of 200 Pa. Ten readings were recorded for each sample.

The filtration performance of the samples was examined by utilizing an air filtration device (TOPAS AFC-131, Frankfurt, Germany). All the samples were cut into a circular sample with a diameter of 17 cm, and the filtration efficiency to DEHS (Diisooctyl sebacate) aerosol particles was obtained under the airflow rate of 18 m^3^/h. The diameter of the DEHS aerosol particles ranged from 0.225 μm to 7.25 μm.

### 2.6. Other Characterization Methods

The morphology and microstructure of the PPS NPFF and the decorated Mn/Ce oxides catalysts were observed by using a scanning electron microscopy (SEM, Phenom Pro SEM, Hitzacker, Germany), and a field emission scanning electron microscopy (JSM-7800F, JEOL, Tokyo, Japan). The samples were subjected to gold sputter-coating for 60 s in a vacuum (SBC-12, KYKY Technology Co., Ltd., Beijing, China) prior to imaging. A X-Max Energy-Dispersive X-ray spectrometer (JEOL Ltd., Tokyo, Japan) was coupled to SEM for the elemental analysis of the sample. Surface areas and pore diameters on the sample fibers were characterized on a fully automated Quantachrome Autosorb iQ3 (Orlando, FL, USA) surface area analyzer using nitrogen adsorption-desorption. The specific surface areas were calculated based on the Brunauer-Emmett-Teller (BET) method and the pore diameters were estimated by using the Barrett-Joyner-Halenda (BJH) method. The pore size distribution and average pore size of the whole sample were determined by using an aperture tester (TOPAS PSM-165, Frankfurt, Germany) with a constant testing area of 2.01 cm^2^. All data were tested and acquired from 5 set of specimens. The mechanical characterization of all samples was performed by a universal testing machine (3382, Instron, Boston, MA, USA). The sample size was 40 mm length × 20 mm width. For each sample, the distance between the two clamps, clamp speed and test volume were fixed at 20 mm, 5 mm/min and 5 times, respectively.

## 3. Results and Discussion

### 3.1. Catalyst Loading Efficiency and Stability

**Figure 3:**
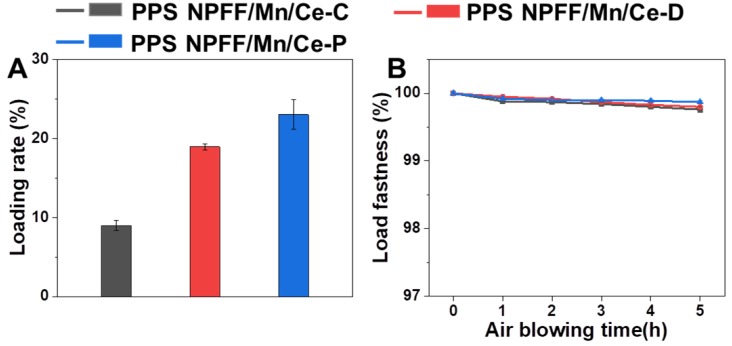
(**A**) Catalyst loading rate and (**B**) catalyst loading fastness of the three Mn/Ce oxides decorated PPS NPFF prepared by different methods.

Figure 3 shows the effects of different pretreatment methods on the loading efficiency and stability of Mn/Ce-based catalysts decorated on the PPS NPFF. The results presented in Figure 3A displayed that the Mn/Ce oxides decorated PPS NPFF prepared with different methods afforded different catalyst loading rates. In general, both PPS NPFF/Mn/Ce-D and PPS NPFF/Mn/Ce-P prepared from the two different pretreatment methods exhibited obviously enhanced catalyst loading rates compared with the PPS NPFF/Mn/Ce-C without any pretreatments. After systematic optimization (as shown in Supplementary Information), the maximum catalyst loading rate pretreated by SA deposition and plasma treatment could reach to 18.95% and 23.03%, respectively. The loading rate of PPS NPFF/Mn/Ce-P was demonstrated to be the highest, which may be due to the notably improved number of active groups on the PPS NPFF surface produced by low temperature plasma treatment.

Catalyst loading fastness is another important indicator for evaluating the catalyst loading status on the surface of PPS NPFF. Figure 3B indicates that all three Mn/Ce oxides decorated PPS NPFF exhibited great catalyst loading stability. With the air blowing time increasing, the fastness of the catalyst decreased slightly. After 5 h of air blowing, the loading fastness was only slightly different from that of the original sample. Moreover, the loading fastness of PPS NPFF/Mn/Ce-P was higher than those of the other two groups, i.e., PPS NPFF/Mn/Ce-D and PPS NPFF/Mn/Ce-C, and the loading fastness of the PPS NPFF/Mn/Ce-P group still maintained 99.8% after 5 h of air blowing, which demonstrated that the low temperature plasma treatment could significantly improve adhesion between the Mn/Ce catalyst and the PPS NPFF.

### 3.2. Morphology and Elemental Analysis

**Figure 4:**
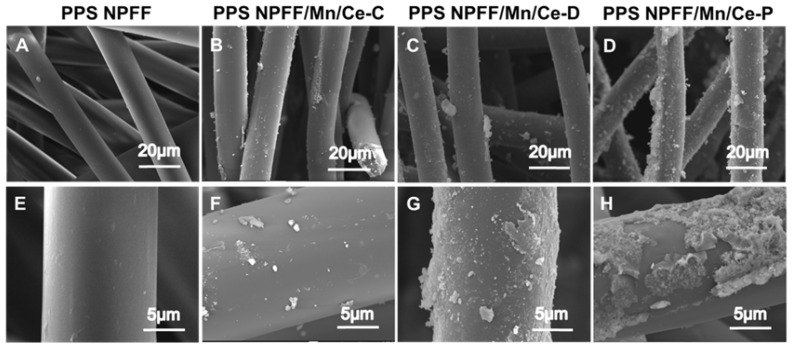
(**A**–**D**) Scanning electron microscopy (SEM) images and (**E**–**H**) FE-SEM (Field emission scanning electron microscopy) images of the original PPS NPFF and the three different Mn/Ce oxides decorated PPS NPFF.

Figure 4A,E shows that the fibrous structure with smooth fiber morphology was observed for the original PPS NPFF without any processing. In comparison, the Mn/Ce oxides decorated PPS NPFF exhibited significantly different morphology. Figure 4B,F shows the morphological images of PPS NPFF/Mn/Ce-C with different magnifications. The Mn/Ce oxides were less developed in this case, since the poor interaction between the Mn/Ce particles and the fibers of PPS NPFF was formed. Figure 4C,G displayed that the Mn/Ce oxides were better dispersed on the fiber surface of PPS NPFF after the pretreatment of SA deposition. SA was firstly deposited on the fiber surface, thereby providing more active sites for the consolidation of Mn/Ce-based catalysts. The topographical images of the PPS NPFF/Mn/Ce-P (Figure 4D,H) presented that obviously increased Mn/Ce oxides were found on the fiber surface, and it had a certain degree of agglomeration in some areas. The low temperature plasma treatment could improve surface roughness, as well as produce more active groups, thereby resulting in the enhanced adhesion between the catalyst and the PPS NPFF. These results suggested that the pretreatment methods had a positive effect on the presence of catalyst particles on the fiber surface of PPS NPFF. In addition, the PPS NPFF/Mn/Ce-P pretreated by low temperature plasma treatment had been demonstrated to exhibit great catalyst dispersion on the surface of PPS NPFF.

**Figure 5:**
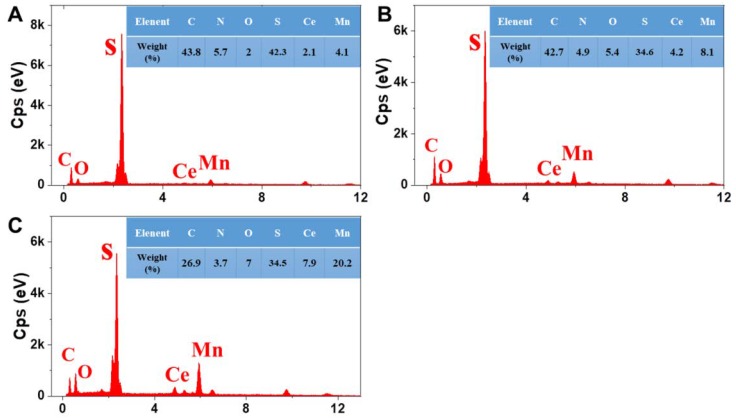
EDS (X-Max Energy-Dispersive X-ray spectrometer) images of the three different Mn/Ce oxides decorated PPS NPFF: (**A**) PPS NPFF/Mn/Ce-C; (**B**) PPS NPFF/Mn/Ce-D; (**C**) PPS NPFF/Mn/Ce-P.

EDS(X-Max Energy-Dispersive X-ray spectrometer)was further employed to examine the surface composition and element distribution of the PPS NPFF after the catalyst loading. Figure 5A–C shows that all the three different Mn/Ce oxides decorated PPS NPFF were found to contain N, Mn, Ce, S, C, and O elements. Moreover, the plasma-pretreated samples (Figure 5C) exhibited the highest Mn and Ce contents than those of the other two samples (Figure 5A,B), which was consistent with the results shown in Figure 4.

### 3.3. Surface Area and Pore Diameter Analysis

**Figure 6:**
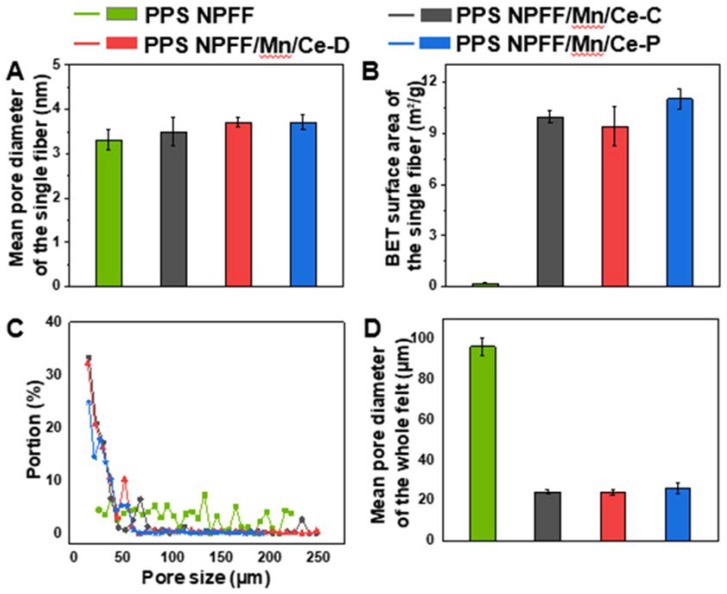
(**A**) Mean pore diameter and (**B**) Brunauer-Emmett-Teller (BET) surface area of the single fiber for the original PPS NPFF and the three different Mn/Ce oxides decorated PPS NPFF; (**C**) pore size distribution and (**D**) mean pore diameter of the whole fibrous felt for the original PPS NPFF and the three different Mn/Ce oxides decorated PPS NPFF.

Figure 6A shows the testing results of mean pore diameter on the fiber surface. The mean pore diameters of original PPS NPFF, PPS NPFF/Mn/Ce-C, PPS NPFF/Mn/Ce-D, and PPS NPFF/Mn/Ce-P were determined to be 3.31 nm, 3.501 nm, 3.707 nm, and 3.706 nm, respectively. The surface area of the fibers was apparently different, as can be seen from Figure 6B. The original PPS NPFF has the lowest value of BET surface area (0.207 m^2^/g). In comparison, the BET surface area of the three different Mn/Ce oxides decorated PPS NPFF increased significantly, which may be due to the good dispersion of Mn/Ce particles on the PPS fiber surface. In the case of PPS NPFF/Mn/Ce-P, the BET surface area was greatly increased to 11.029 m^2^/g, most likely due to the abundant deposit of catalyst on the surface of the fiber. The obviously increased surface area may provide more space, which could favor the interaction between the catalyst and reaction gas.

In spite of the pore diameter of the single fibers, we also tested the pore diameter distribution of the whole fibrous felt as shown in Figure 6C. The mean pore diameter of the whole fibrous felt is calculated in Figure 6D. Notably, the original PPS NPFF presented a wider pore diameter distribution compared with the three different Mn/Ce oxides decorated PPS NPFF. The original PPS NPFF possessed the highest value of mean pore diameter (94.37 μm), whereas all the mean pore diameters of three different Mn/Ce oxides decorated PPS NPFF reduced to below 30 μm. Taken together, the catalyst loading could significantly narrow the pore diameter distribution (Figure 6C) and obviously decrease the value of the mean pore diameter (Figure 6C).

### 3.4. Mechanical Property

**Figure 7:**
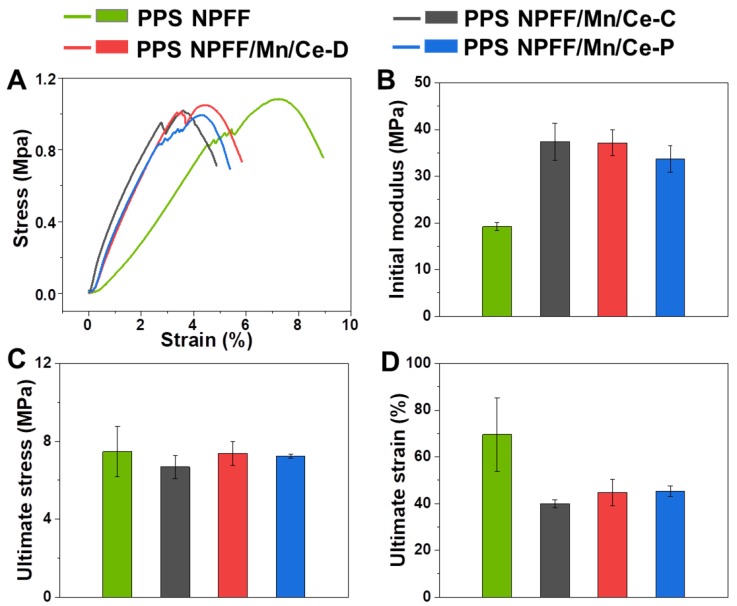
(**A**) Representative stress-strain curves, (**B**) initial modulus, (**C**) ultimate tensile stress, and (**D**) ultimate strain of the original PPS NPFF and the three different Mn/Ce oxides decorated PPS NPFF.

Uniaxial tensile testing results of the original PPS NPFF and the three different Mn/Ce oxides decorated PPS NPFF are shown in Figure 7. Typical stress-strain curves in Figure 7A indicated that all the tested samples exhibited similar mechanical behavior characteristics. In comparing the three different Mn/Ce oxides decorated PPS NPFF with the original PPS NPFF, they presented obviously higher initial modulus (Figure 7B), but relatively lower breaking stress and strain (Figure 7C,D), attributed to the necessary thermal treatment experienced during the catalyst loading process. The three different Mn/Ce oxides decorated PPS NPFF still possessed excellent mechanical properties to satisfy the requirements for both dust removal and denitration application.

### 3.5. Filtration Property and Air Permeability

**Figure 8:**
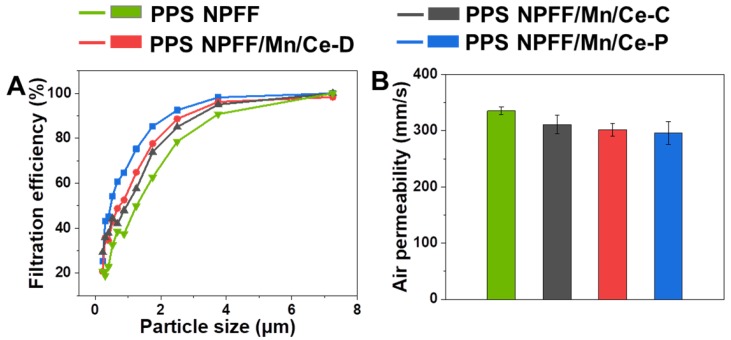
(**A**) Filtration efficiency and (**B**) air permeability of the original PPS NPFF and the three different Mn/Ce oxides decorated PPS NPFF.

We performed filtration experiments on all PPS NPFF with or without further processing. Figure 8A comparatively depicts the grade efficiency of each sample for fine dust in the regime of 0.225 μm to 7.25 μm. The same profile pattern can be observed in all instances, i.e., the filtration efficiency increases with increasing particle size. Relatively low capture efficiency was found for the original PPS NPFF. As expected, the catalyst loading process could improve the overall filtration performance, showing a much higher capture efficiency than that of the original PPS NPFF. This observation is ascribed to the significantly reduced pore diameter distribution and mean pore diameter, caused by the catalyst loading process. The PPS NPFF/Mn/Ce-P showed the highest efficiency among all the samples, indicating the advantages of applying the low temperature plasma treatment on the PPS NPFF modification. In practice, all the three different Mn/Ce oxides decorated PPS NPFF, i.e., PPS NPFF/Mn/Ce-C, PPS NPFF/Mn/Ce-D, PPS NPFF/Mn/Ce-P, could perform well when target particles were larger than 4 μm. It is also observed in Figure 8B that the catalyst loading process did not affect the overall air permeability of the three different Mn/Ce oxides decorated PPS NPFF, presenting slightly reduced air permeability compared with the original PPS NPFF.

### 3.6. Denitration Mechanism and Performance

**Figure 9:**
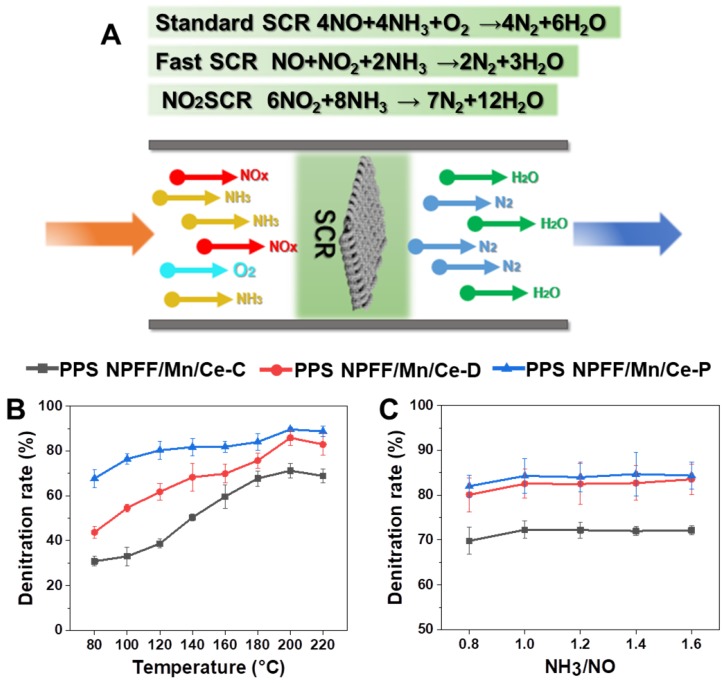
(**A**) Schematic model of the denitration mechanism by using our denitration; denitration rate for the three different Mn/Ce oxides decorated PPS NPFF as a function of (**B**) operating temperature and (**C**) NH_3_/NO flow ratio.

The general reactions involved in NH_3_-SCR are shown in Figure 9A. NH_3_ was employed as a reducing agent to reduce harmful NOx to harmless N_2_ and H_2_O under the synergetic action with O_2_. There were three general reactions, which were called standard SCR, fast SCR, and NO_2_ SCR reactions [7,32]. The catalyst performance was found to be a dominant factor for the above-mentioned reactions [11,33]. In the present study, we employed two pretreatment methods including SA deposition and plasma treatment to modify the PPS NPFF before the impregnation process to help improve the catalyst loading rate and stability, which was expected to be conducive for the NH_3_-SCR denitration activity. Figure 9B,C shows the effect of different preparation methods on the denitration activity of finally-obtained Mn/Ce oxides decorated PPS NPFF. Figure 9B indicates that the Mn/Ce oxides decorated PPS NPFF prepared with three different methods imparted different catalytic activities in the temperature range of 80 °C to 220 °C. In general, the PPS NPFF/Mn/Ce-D and PPS NPFF/Mn/Ce-P which both experienced pretreatment exhibited better catalytic activity and presented higher denitration rate, compared to PPS NPFF/Mn/Ce-C without pretreatment. The same pattern can be observed in all instances, i.e., the denitration rate increased with increasing temperature from 80 °C to 200 °C. The denitration rate reached the optimum value at 200 °C. When the temperature exceeded 200 °C, the denitration rate decreased slightly. Importantly, it can be concluded that PPS NPFF/Mn/Ce-P prepared by low temperature plasma treatment exhibited the highest denitration rate, and the denitration rate reached more than 80% at 220 °C. The previous studies have also demonstrated that the reaction temperature played a determined role on the denitration rate of the Mn/Ce catalyst [34,35].

From the three mentioned reactions in Figure 9A, we could figure out the imposed quantity of NH_3_ as another key factor which could significantly affect the complete reduction of NOx emissions. Figure 9C displays that how the NH_3_/NO flow ratio affects the denitration rate of all the Mn/Ce oxides decorated PPS NPFF prepared by three different methods. We found that the denitration rate increased with the NH_3_/NO flow ratio increasing when the NH_3_/NO flow ratio was below 1. The denitration rate significantly improved when the NH_3_/NO flow ratio reached 1, and it tended to be stable when the NH_3_/NO flow ratio exceeded 1.

## 4. Conclusions

A series of Mn/Ce oxides decorated PPS NPFF were prepared by using three different methods. Two pretreatment methods, i.e., SA deposition and plasma treatment, were demonstrated to effectively modify the PPS NPFF, which were beneficial for improving the catalyst loading rate and stability of Mn/Ce oxides. In all, the two different pretreatment methods could impart the eventually-obtained Mn/Ce oxides decorated PPS NPFF with great dust removal activity as well as excellent denitration outcomes. This study provides a theoretical and experimental basis for the denitrification-dust removal integration process, and is of great significance for simultaneous removal of both fine dust and NOx in the exhaust emissions.

## Supplementary Materials

The following are available online at https://www.mdpi.com/2073-4360/12/1/168/s1, Figure S1: Loading rate under each parameter of the impregnation and heat treatment process: (A) Ce molality; (B) Mn/Ce molar ratio of impregnation solution; (C) Impregnation time. Figure S2: Loading rate under each parameter of SA deposition: (A) The mass fraction of SA; (B) Immersion time of SA deposition. Figure S3: Loading rate under each parameter of plasma treatment: (A) Voltage; (B) Duty ratio; (C) Jet moving speed; (D) Frequency.
