# Supplementary material for: Mn/Ce Oxides Decorated Polyphenylene Sulfide Needle-Punching Fibrous Felts for Dust Removal and Denitration Application
Source: Polymers (Basel). 2020 Jan 9;12(1):168. doi: 10.3390/polym12010168 (PMC7022841; doi:10.3390/polym12010168)
Supplement: Supplementary file 1 [file polymers-12-00168-s001.pdf]

Supplementary Materials

Mn/Ce Oxides Decorated Polyphenylene Sulfide Needle-Punching Fibrous Felts for Dust Removal and Denitration Application

Ying Chen [1,†], Hongwei He [1,†], Shaohua Wu [2], Xin Ning [1], Fuxing Chen [1], Yanru Lv [1], Juan Yu [1] and Rong Zhou [1] *

[1] Industrial Research Institute of Nonwovens & Technical Textiles, College of Textiles & Clothing, Qingdao University, Qingdao 266071, China; cheny06301@163.com (Y.C.); hhwpost@163.com (H.H.) xning@qdu.edu.cn (X.N.) fuxing1991@gmail.com (F.C.) lyanru_777@163.com (Y.L.); yujuan_2018@126.com (J.Y.)

[2] College of Textiles & Clothing, Qingdao University, Qingdao 266071, China; shaohua.wu@qdu.edu.cn

* Correspondence: rzhouqdu@126.com

†These two authors contributed equally to this work.

This material includes:

1.Single parameters optimization of impregnation and thermal treatment processes

2.Single parameters optimization of SA deposition.

3. Orthogonal experiments for parameters optimization of plasma treatment

1. Single parameters optimization of impregnation and thermal treatment processes

In the experimental process of impregnation, the main conditions affecting the experimental results are: Ce molality, Mn/Ce molar ratio of impregnation solution, impregnation time, temperature and time of thermal treatment. When exploring a single factor, make sure that the other conditions are the same and only change one experimental factor. The loading rate of the catalyst-decorated PPS NPFF was used as an evaluation standard. **Figure S1** shows the loading rate of the catalyst under different parameters of impregnation.

The loading rate of the PPS NPFF increases with the increase of the Ce molality. From **Figure S1**A, when the Ce molality is 0.07 mol/L, the loading rate reaches the highest value. However, the higher the Ce molality, the higher the cost of loading catalyst, and the other properties of PPS NPFF may be affected. The optimal Ce molality was found to be 0.07 mol/L.

Figure S1B indicated that when the molar ratio of Mn/Ce is 6/1, the synergistic effect between the two active components of manganese dioxide and cerium oxide is enhanced, and the activity of the catalyst is obviously improved. The optimal Mn/Ce molar ratio of impregnation solution was found to be 6/1.

The immersion solution enters the PPS NPFF with capillary action. After 60 min, the immersion solution in PPS NPFF was saturated and the loading rate did not continue to increase. The results presented in **Figure S1**C that the optimal immersion time of impregnation was 60 min.

In addition, Mn $(NO_3)_2$ decomposes into MnO_2 at 160 ° C -200 ° C, and Ce $(NO_3)_3$ decomposes to CeO_2 at temperatures above 200 °C. As the temperature increases, the energy consumption increases greatly, and the service life of the fabric also decreases. In order to allow both Mn $(NO_3)_2$ and Ce $(NO_3)_3$ to decompose into oxides at high temperatures, while increasing the service life, the optimum calcination temperature of thermal treatment was 200 °C. Similarly, time of thermal treatment directly affects the amount of active components of the catalyst. If the time is too short, the calcination process is insufficient and the active component of the catalyst cannot be completely decomposed.

Excessive time will also cause waste of energy. After literature investigation and experience, we selected the time of thermal treatment was 60min.

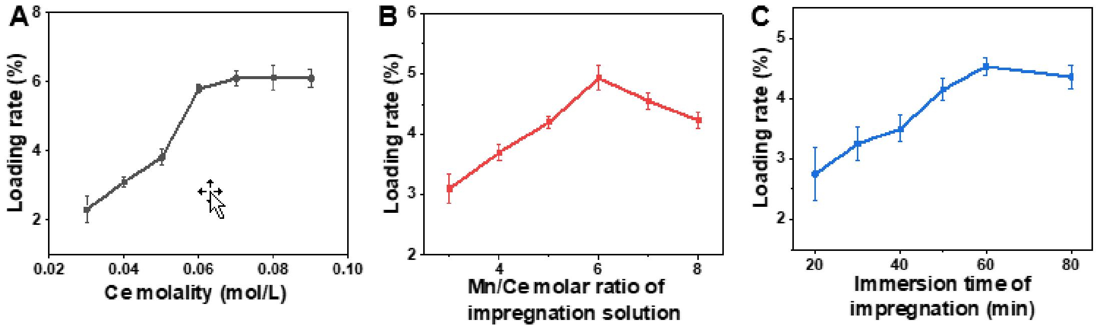

Figure S1. Loading rate under each parameter of the impregnation and heat treatment process: (A) Ce molality; (B) Mn/Ce molar ratio of impregnation solution; (C) Impregnation time.

2. Single parameters optimization of SA deposition

SA is slightly soluble in water and is almost saturated when the mass fraction is 2.0%. See from **Figure S2**A, when directly impregnated without SA, the loading rate of the catalyst is extremely low, only about 3%. Moreover, as the mass fraction of SA increases, the loading rate also increases gradually. When the mass of SA increased to 0.8%, the loading rate no longer increases and there is a downward trend. The optimal mass fraction of SA was 0.8%.

When the immersion time is insufficient, the immersion solution cannot be completely loaded on the PPS NPFF, which may cause insufficient utilization of the immersion solution. The results presented in **Figure S2**B that after the immersion time exceeds 60 min, the catalyst loading rate hardly increases, so the optimal immersion time was 60 min.

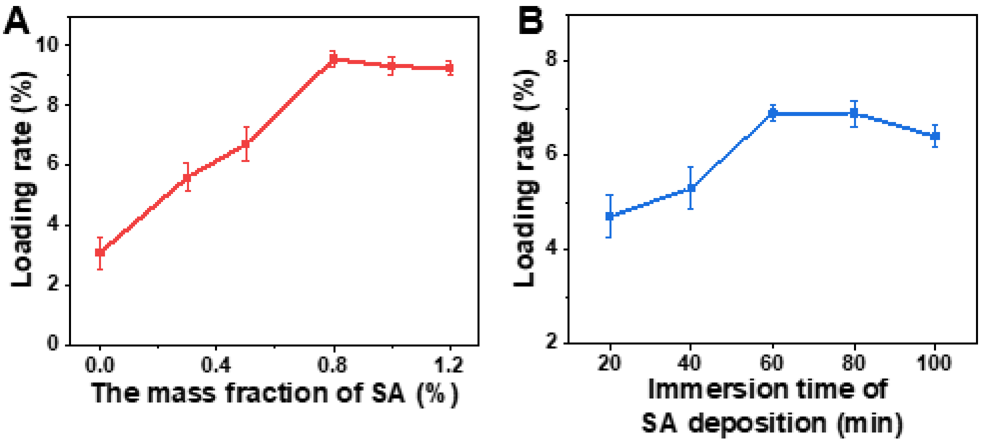

Figure S2. Loading rate under each parameter of SA deposition: (A) The mass fraction of SA; (B) Immersion time of SA deposition.

3. Orthogonal experiments for parameters optimization of plasma treatment

In the experimental process of plasma treatment, the setting of plasma parameters has a great influence on the results. The main parameters affecting the experimental results are: voltage, duty ratio, jet moving speed and frequency. When exploring a single factor test, make sure that the other conditions are the same and only change one experimental factor. The loading rate of the catalyst was used as a standard for the evaluation results. **Figure S3** shows the results of each single factor.

The discharge phenomenon occurs when the voltage exceeds 250V, and the catalyst loading rate of PPS increases first and then decreases with the increase of voltage (**Fig. S3**A); The duty ratio is the ratio of each discharge pulse stalled between one pulse cycle. A reasonable duty ratio allows the

energy generated by each pulse to be fully utilized during duty cycles. When the duty ratio is too high, the discharge is uneven, which affects the sustainability and stability of the active particles, resulting in a decrease in the loading rate (**Fig. S3**B); For the same sample, the higher the jet moving speed, the shorter the jet contact time with the sample and the lower the energy output (**Fig. S3**C); The frequency is an important factor affecting the input energy in the plasma reaction process. The increase of the frequency can increase the number of pulse discharges per unit time, resulting in the enhancement of the surface etching and the increase of the bonding performance of the felt (**Fig. S3**D).

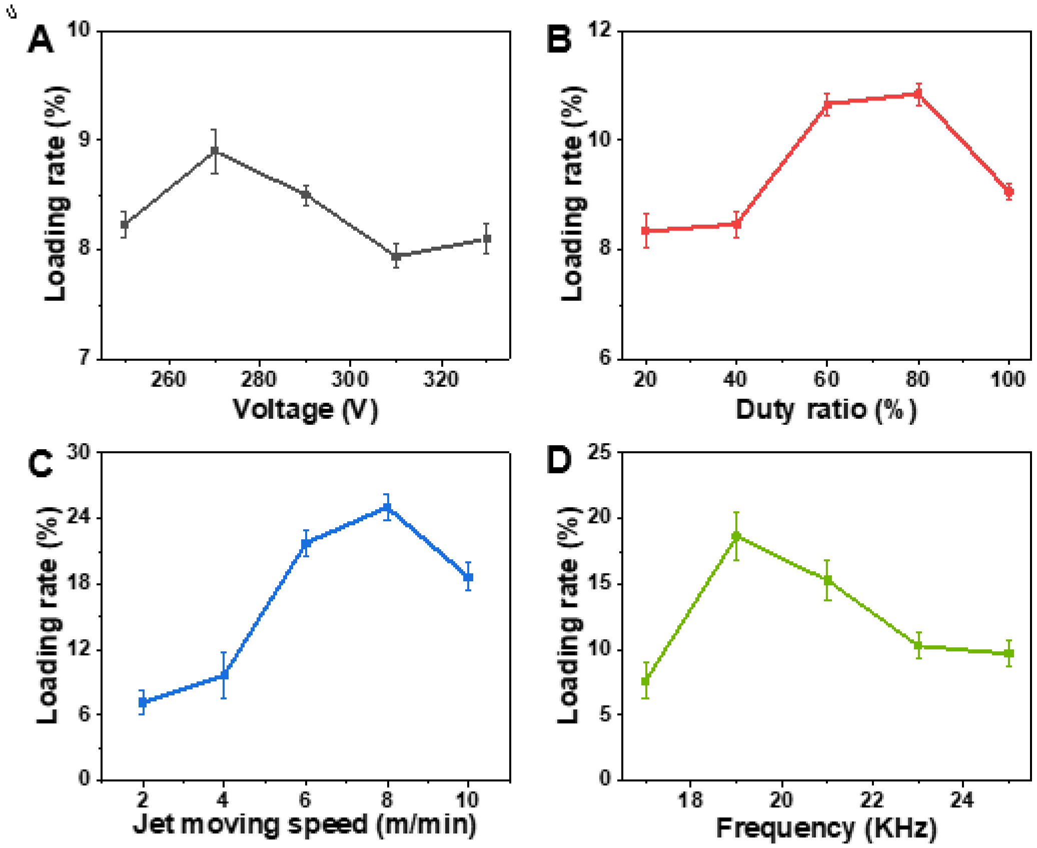

Figure S3. Loading rate under each parameter of plasma treatment: (A) Voltage; (B) Duty ratio; (C) Jet moving speed; (D) Frequency.

In view of the above single factor test results, the factors and levels of the orthogonal experiment were selected as shown in **Table S1**.

Table S1. Experimental factors and levels of impregnation methods.

**Levels**	**Factors**			
	**A**	**B**	**C**	**D**
	**Voltage (V)**	**Duty ratio (%)**	**Jet moving speed (m·min^{-1})**	**Frequency (KHz)**
1	270	60%	6	19
2	280	70%	7	20
3	290	80%	8	21

The L9 (3^4) orthogonal experiment table was selected according to the test factors and the levels to arrange the experiment. The obtained orthogonal experiment table is shown in **Table S2**.

From the results of the orthogonal test, the loading rate is the highest when the voltage is 280V (A2). The duty ratio has a large influence on the sample loading rate. And the loading rate average value is reached 25.790 when the duty ratio is 60% (B1). From the average value, the load rate is the highest When the jet moving speed is 8 m/min (C3). Obviously, when the frequency is 19 KHz, the

loading rate is the highest (D1). The sequence of the factors was determined by orthogonal experiments: duty ratio, frequency, voltage, jet moving speed.

Therefore, the optimum conditions for plasma treatment of the supported catalyst are: voltage 280V, duty ratio 60%, jet moving speed 8m/min, frequency 19KHz.

Table S2. L9 (3^4) Orthogonal experiment table of impregnation methods.

**Test number**	Factors				Load rate (%)
	A	B	C	D	
1	1	1	1	1	18.64
2	1	2	2	2	18.86
3	1	3	3	3	25.23
4	2	1	2	3	26.82
5	2	2	3	1	22.92
6	2	3	1	2	16.24
7	3	1	3	2	16.94
8	3	2	1	3	16.15
9	3	3	2	1	17.9
Average 1	20.910	**25.790**	17.010	**22.733**	
Average 2	**21.993**	19.911	21.193	17.347	
Average 3	16.997	19.790	**21.697**	19.820	
Range	4.996	6.001	4.687	5.386	
Primary and secondary order	B>D>A>C				
Optimal levels	A2	B1	C3	D1	
